# A Combined Histone Deacetylases Targeting Strategy to Overcome Venetoclax Plus Azacitidine Regimen Resistance in Acute Myeloid Leukaemia: Three Case Reports

**DOI:** 10.3389/fonc.2021.797941

**Published:** 2021-12-09

**Authors:** Bin-Ru Wang, Chao-Ling Wan, Song-Bai Liu, Qiao-Cheng Qiu, Tian-Mei Wu, Jun Wang, Yan-Yan Li, Shuai-Shuai Ge, Yan Qiu, Xiang-Dong Shen, Sheng-Li Xue, Zheng Li

**Affiliations:** ^1^ National Clinical Research Center for Hematologic Diseases, Jiangsu Institute of Hematology, The First Affiliated Hospital of Soochow University, Suzhou, China; ^2^ Institute of Blood and Marrow Transplantation, Collaborative Innovation Center of Hematology, Soochow University, Suzhou, China; ^3^ Suzhou Key Laboratory of Medical Biotechnology, Suzhou Vocational Health College, Suzhou, China

**Keywords:** venetoclax (BCL2 inhibitor), chidamide, histon deacetylase inhibitors (HDACi), R/R acute myeloid leukemia AML, targeted therapy

## Abstract

The management of patients with relapsed or refractory (R/R) acute myeloid leukaemia (AML) remains a challenge with few reliably effective treatments. Chidamide, a new selective HDAC inhibitor, has demonstrated some effectiveness in AML patients. Herein, we reported three patients with R/R AML who were unresponsive to venetoclax plus azacitidine (VA) but were successfully treated with VA when chidamide was added to the regimen. MCL1 is one of the anti-apoptotic proteins. Chidamide targets the MCL1 protein, which may permit venetoclax resistance when upregulated. We determined MCL1 protein expression in different AML cell lines, and chidamide could downregulate MCL1 expression in venetoclax resistance AML cells. In general, our experience showed that the chidamide/VA combination could improve the condition of R/R AML patients who are resistant to VA. Formally evaluating this regimen in R/R AML patients may be meaningful.

## Introduction

AML is a disease caused by the blocked differentiation of myeloid haematopoietic stem cells and the clonal proliferation of primitive or immature myeloid cells in the bone marrow (BM). With current chemotherapies, approximately 10% to 40% of newly diagnosed AML patients cannot achieve complete remission (CR), and more than 50% of AML patients will ultimately relapse (1). Although higher clinical responses may be achieved with salvage intensive chemotherapy, durability is generally short lived and counterbalanced by higher toxicities, especially for elderly individuals with poor performance status.

According to the NCCN guidelines, the BCL2 inhibitor venetoclax combined with hypomethylating agents or low-dose cytarabine represents an important new therapy not only for treatment-naïve elderly AML patients but also for relapsed or refractory AML patients deemed ineligible for intensive therapy (2, 3). However, with advances in the clinical application of venetoclax-based combination therapy, primary resistance and clonal evolution leading to adaptive resistance to venetoclax have become a major hindrance for AML treatment (4). Therefore, exploring new combination regimens to overcome drug resistance is urgently needed.

Here, we present three relapsed AML patients who were unresponsive to the venetoclax plus azacitidine (VA) regimen and were successfully salvaged by chidamide in combination with venetoclax and azacitidine. The regimen was safe and effective. This may provide a new choice for relapsed or refractory AML patients with resistance to VA.

## Case Presentation

**Figure 1:**
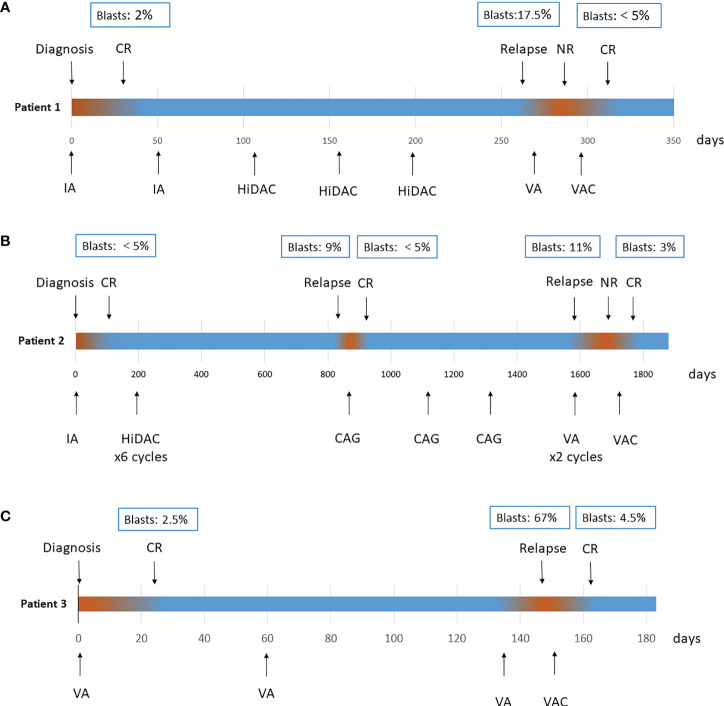
Treatment process of patient 1 **(A)**, patient 2 **(B)** and patient 3 **(C)**. CR, complete remission; IA, cytarabine 100 mg/m^2^ continuous infusion day1–7, idarubicin 12 mg/m^2^ day1–3; HiDAC, cytarabine 2 g/m^2^ over 3 h every 12 h on day1–3; CAG, cytarabine 10 mg/m^2^ every 12 h, day1–14; aclarubicin 5–7 mg/m^2^, daily on day1–8; and concurrent use of G-CSF 200 µg/m^2^/day; VA, venetoclax once daily (100 mg day1, 200 mg day2, 400 mg day3–28) and azacitidine 75 mg/m^2^ day1–7; VAC, venetoclax once daily (100 mg day1, 200 mg day2, 400 mg day3–21), azacitidine 75 mg/m^2^ daily day1–7, chidamide 5 mg daily day1–7.

Patient 1: A 65-year-old male was diagnosed with AML in January 2020 with the manifestation of leukocytosis, thrombocytopenia and anaemia. BM aspiration revealed a hypercellular BM with 50% blasts. Flow cytometry showed the immunophenotype of myeloid blasts. Cytogenetics revealed a complex karyotype, and a molecular panel identified aberrations in *ASXL1*, *CEBPA*, *JAK2*, *and RUNX1*. None of the 41 gene fusions were detected by using multiple RT–PCR assay (5). Therefore, AML with adverse risk was diagnosed according to genetic risk stratification (6). After one course of induction treatment with the IA regimen [idarubicin 12 mg/m^2^ day1-3, cytarabine 100 mg/m^2^ continuous infusion day1-7.], the patient achieved complete remission with minimal residual disease as low as 5.8x10^-4^ by flow cytometry analysis. Genetic analyses showed that all gene mutations were negative. Subsequently, the patient refused bone marrow transplant for financial reasons. He received one course of the IA regimen and 3 courses of the high-dose Ara-c (HiDAC) regimen [cytarabine 2 g/m^2^ over 3 h every 12 h on day1–3.] as consolidation therapies. However, the remission duration only lasted for 11 months. In November 2020, relapsed BM morphology was detected, with 17.5% blasts concurrent with molecular aberration recurrence. Therefore, relapsed AML was diagnosed. Initially, venetoclax combined with azacitidine [VA, venetoclax once daily (100 mg day1, 200 mg day2, 400 mg day3-28) and azacitidine 75 mg/m^2^ day1-7.] was administered as a salvage therapy, an effective regimen recommended for the treatment of R/R AML patients who are ineligible for intensive salvage chemotherapy, but progressive disease was observed. Then, his treatment plan switched to a chidamide combined with venetoclax plus azacitidine regimen [chidamide 5 mg daily day1-7, venetoclax 100 mg day1, 200 mg day2, 400 mg day3-21; azacitidine 75 mg/m^2^ daily day1-7.], and CR was achieved after one course of therapy. The patient treatment process is shown in **Figure 1A**. After 3 months of follow-up, unfortunately, the patient gave up further treatment and passed away due to disease progression. The overall survival time was 1.5 years from first diagnosis.

Patient 2: A 57-year-old male was diagnosed with AML in July 2016. Investigation revealed a pancytopenia. BM examination showed a hypercellular marrow with 65% myeloid blasts. Flow cytometry analysis showed the immunophenotype of myeloid blasts. Cytogenetics revealed del (7) (q22q36), and a molecular panel identified aberrations in *DNMT3A* and *IDH2*. The patient achieved CR after 1 cycle of the IA regimen [idarubicin 12 mg/m^2^ day1-3, cytarabine 100 mg/m^2^ continuous infusion day1-7.] and received 6 courses of the HiDAC regimen [cytarabine 2 g/m^2^ over 3 h every 12 h on day1–3.] as consolidation therapies while not adopting allogeneic haematopoietic stem cell transplantation. The patient experienced his first relapse 2 years after first remission with a 9% immature cell level in the BM and was treated with the CAG [cytarabine 10 mg/m^2^ every 12 h, day1-14; aclarubicin 5-7 mg/m^2^, daily on day1-8; and concurrent use of G-CSF 200 µg/m^2^/day.] regimen for 3 cycles, resulting in a second CR in March 2019. A second relapse occurred 20 months later. The patient began the VA regimen [venetoclax once daily (100 mg day1, 200 mg day2, 400 mg day3-28) and azacitidine 75 mg/m^2^ day1-7.], but no response was observed after 2 courses of therapies. Finally, the patient received a chidamide combined with venetoclax plus azacitidine regimen [chidamide 5 mg daily day1-7, venetoclax 100 mg day1, 200 mg day2, 400 mg day3-21; azacitidine 75 mg/m^2^ daily day1-7.] as salvage therapy as described above. The patient achieved his third CR. Treatment process is shown in **Figure 1B**. There were no infectious complications observed with the combination, and the duration of neutropenia was 10 days. After 1 month of follow-up, the patient remains in CR and is preparing for transplantation.

Patient 3: A 60-year-old female was diagnosed with AML in December 2020. BM examination showed a hypercellular marrow with 32% myeloid blasts. A molecular panel identified aberrations in *RUNX1*. Karyotype was normal. All patient baseline characteristics at diagnosis and treatment characteristics are shown in **Supplementary Table 1**. For induction therapy, the patient received the VA [venetoclax once daily (100 mg day1, 200 mg day2, 400 mg day3-28) and azacitidine 75 mg/m^2^ day1-7.] regimen and achieved CR after one course. Subsequently, she continued two courses of VA as consolidation therapy, but progressive disease was observed during the second course, with 67% blasts in BM. Then, a chidamide combined with venetoclax plus azacitidine regimen [chidamide 5 mg daily day1-7, venetoclax 100 mg day1, 200 mg day2, 400 mg day3-21; azacitidine 75 mg/m^2^ daily day1-7.] was given. After one course, the patient obtained CR. Treatment process is shown in **Figure 1C**. During the whole course, no severe adverse events occurred. After 1 month of follow-up, the patient remains in CR at the time of writing.

## Discussion

Venetoclax, a newly orally available and selective BCL2 inhibitor, has shown great effectiveness in various haematologic malignancies, with less thrombocytopenia observed (7). Recently, venetoclax in combination with hypomethylation agents or cytarabine has been approved by the Food and Drug Administration (FDA) for the treatment of patients with newly diagnosed AML unfit for intensive chemotherapy (3, 8). However, resistance to venetoclax can be acquired through the upregulation of anti-apoptotic proteins in the BCL2 family, such as myeloid cell leukaemia 1 (MCL1) and BCL2-like 1 (BCL2L1, also known as BCL-XL) (9). The overexpression of the anti-apoptotic proteins MCL1 and BCL-XL blocks the activation of BAX/BAK by binding to BH3-only proteins, thereby blocking cell apoptosis and eventually causing disease relapse (10, 11). Therefore, targeting only BCL2 often fails to achieve the expected therapeutic effect. The combination of specific drugs with venetoclax to alter the balance between pro-apoptotic and anti-apoptotic proteins may be a clinically optional treatment strategy for overcoming venetoclax resistance (12).

MCL1 is one of the most frequently amplified genes in both solid and haematologic malignancies. In particular, elevated expression of MCL1 confers resistance to BCL2/BCLXL inhibitors (13). Jordan et al. demonstrated that AML patients with monocytic disease are more likely to be refractory to venetoclax-based regimens because monocytic AML reduces the expression of venetoclax-targeting BCL2 while preferentially relies on MCL1 for energy metabolism and survival (14). However, selective MCL1 inhibitors are not clinically available. In the absence of direct inhibitors of MCL1, pharmacologic strategies that indirectly suppress MCL1 activity by diminishing MCL1 protein expression may be an alternative treatment option to improve venetoclax efficacy (15).

Chidamide, a newly designed selective histone deacetylase inhibitor, induces an antitumour effect in various cancers, and it has been approved for the treatment of peripheral T-cell lymphoma by the Food and Drug Administration. Preclinical studies have shown that chidamide exhibits synergistic activity in leukaemia cells when combined with hypomethylating agents or DNA-damaging agents (16). Several clinical trials have explored the clinical benefit of histone deacetylase (HDAC) inhibitors in combination with DNA methyltransferase (DNMT) inhibitors for AML patients (17, 18). *In vitro*, it has been proven that low-dose chidamide enhances the anti-AML activity of venetoclax by downregulating MCL1 and upregulating BIM (15). Given these facts, the patients were given the chidamide regimen combined with venetoclax and azacytidine, and the protocol proved to be effective with tolerable toxicity.

**Figure 2:**
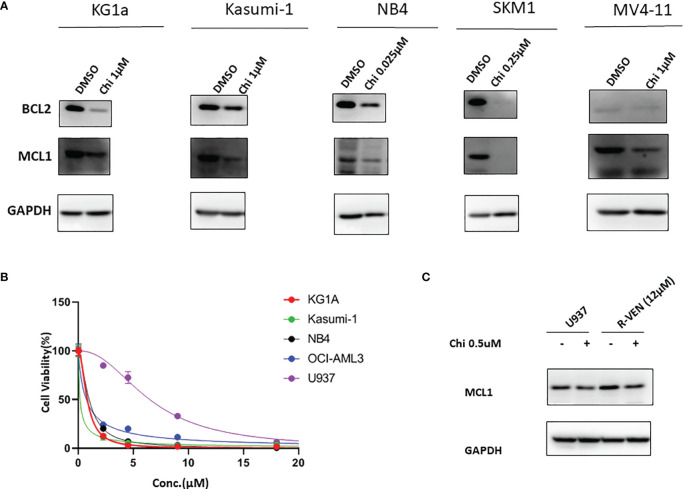
Western blot for BCL2 and MCL1 from chidamide-treated AML cell lines. **(A)**. Effect of chidamide (Chi) treatment on the protein expression of BCL2 and MCL1 in different AML cell lines. All the cell lines were treated with a suitable concentration of chidamide for 72 hours, and GAPDH was used as an internal control. **(B)**. Venetoclax inhibited cell proliferation in different AML cell lines. All AML cell lines were exposed to different concentration of venetoclax (5 µM, 10 µM, 15 µM and 20 µM) for 72 h, and then cell viability was determined by a CCK-8 assay. **(C)**. MCL1 protein levels were detected by western blot after 24 h of chidamide (0.5 µM) treatment or not. R-VEN (12 µM), U937 cell line resistant to 12 µM venetoclax.

MCL1 plays a critical role in cell apoptosis regulation. To further determine that chidamide could downregulate MCL1 and overcome venetoclax resistance, we then evaluated antiapoptotic protein expression in different tested AML cell lines which were purchased from ATCC (Manassas, US). Different AML cell lines represent the AML FAB classifications accordingly. KG1A, Kasumi-1, NB4, and OCI-AML3 and U937, SKM-1, and MV4–11 represent M1, M2, M3, M4 and M5, respectively. As shown in **Figure 2A**, MCL1 was expressed in almost all cell lines, while treatment with chidamide resulted in a decrease in the protein level of MCL1. Venetoclax markedly inhibited the proliferation of different AML cell lines (M1~M5) at low concentrations except U937 (IC50≈6 µM) (**Figure 2B**). Next, we selected the U937 cell line and established the venetoclax-resistant U937 cell line designated R-VEN by gradually increasing the concentration of venetoclax to 12 µM *in vitro*. The experiment showed that the expression of MCL1 protein increased in R-VEN; however, exposure to chidamide led to a modest reduction in MCL1 expression in R-VEN cells (**Figure 2C**). This result raised the possibility that a low dose of chidamide might partially overcome venetoclax resistance in AML cells by downregulating MCL1. Together, these findings suggest that chidamide may induce venetoclax-resistant leukaemia suppression by downregulating MCL1, which could be further exploited in future clinical trials.

## Conclusion

These results suggest that chidamide plus venetoclax and azacitidine can be safely administered to R/R AML patients and show promising efficacy when patients previously failed the VA regimen. Chidamide is an epigenetic regulator targeting HDAC1, 2, 3, 10 that shows a potential role in altering the imbalance of anti- and pro-apoptotic proteins. To the best of our knowledge, this is the first report of implementing a chidamide combined with a venetoclax plus azacitidine regimen and enabling patients to achieve CR after one course. We suggest that this strategy be considered in patients who are resistant to venetoclax and those who are unfit for intensive chemotherapy. Investigating this combination regimen in R/R AML patients may be meaningful in future planned trials.

## Data Availability Statement

The original contributions presented in the study are included in the article/**Supplementary Material**. Further inquiries can be directed to the corresponding authors.

## Ethics Statement

The studies involving human participants were reviewed and approved by Ethics Committee of the First Affiliated Hospital of Soochow University. The patients/participants provided their written informed consent to participate in this study.

## Author Contributions

B-RW and C-LW collected, analyzed data and wrote the manuscript. Q-CQ contributed to methodology and investigation. S-BL and S-LX performed research and interpreted data. T-MW, JW and Y-YL helped in the collection of samples/data. S-SG, YQ, and X-DS performed the experiments. S-LX and ZL conceived and designed the study. All authors contributed to the article and approved the submitted version.

## Funding

This work was supported by the grants from the National Natural Science Foundation of China (grant No. 81970138), Translational Research Grant of NCRCH (grant No. 2020ZKMB05), Jiangsu Province “333” project, Social Development Project of the Science and Technology Department of Jiangsu (Grant No. BE2021649) and Gusu Key Medical Talent Program (grant No. GSWS2019007).

## Conflict of Interest

The authors declare that the research was conducted in the absence of any commercial or financial relationships that could be construed as a potential conflict of interest.

## Publisher’s Note

All claims expressed in this article are solely those of the authors and do not necessarily represent those of their affiliated organizations, or those of the publisher, the editors and the reviewers. Any product that may be evaluated in this article, or claim that may be made by its manufacturer, is not guaranteed or endorsed by the publisher.
