# Supplementary material for: A Combined Histone Deacetylases Targeting Strategy to Overcome Venetoclax Plus Azacitidine Regimen Resistance in Acute Myeloid Leukaemia: Three Case Reports
Source: Front Oncol. 2021 Dec 9;11:797941. doi: 10.3389/fonc.2021.797941 (PMC8695792; doi:10.3389/fonc.2021.797941)
Supplement: Supplementary file 1 [file Table_1.docx]

Supplementary Material

**1** **Supplementary Table 1.** **Patient baseline characteristics at diagnosis and treatment characteristics.**

| Characteristics | Patient 1 | Patient 2 | Patient 3 |
| --- | --- | --- | --- |
| Age/gender | 64/M | 57/M | 60/F |
| WBC (x10^9^/L) | 19.77 | 1.0 | 3.66 |
| Hb (g/L) | 81 | 60 | 81 |
| PLT (x10^9^/L) | 75 | 21 | 218 |
| Blast proportion in bone marrow (%) | 56 | 65 | 32 |
| Immunophenotype | AML | AML | AML |
| Karyotype | 46,X,-Y,?t(15,17),?16p+,+21[1]/46,XY[2] | 46,XY,del(7),(q22q36)[7]/46,XY[3] | 46,XX[20] |
| Fusion gene | Negative | Negative | Negative |
| Molecular abnormality | *ASXL1,CEBPA,JAK2,RUNX1* | *DNMT3A,IDH2* | *RUNX1* |
| Prior treatment | IA/HiDAC/VA | IA/HiDAC/CAG/VA | VA |
| Disease status and burden before VAC regimen | Relapsed(68%) | Relapsed(29.5%) | Relapsed(67%) |
| Courses of reinduction by VAC regimen/result | 1/CR | 1/CR | 1/CR |
| Anemia/grade | Yes/grade 3 | Yes/grade 3 | Yes/grade 3 |
| Neutropenia/duration | Yes/12 days | Yes/10 days | Yes/6 days |
| Febrile neutropenia | Yes | No | No |
| Other toxicities/grade | Pneumonitis/grade 2 | No | Pneumonitis/grade 2 |
| Follow-up and outcome | dead | In continuous CR for 1 month and preparing for allo-HSCT | In continuous CR for 1 month till now under consolidation therapy |

Abbreviations: M, male; F, female; WBC, white blood cell; Hb, haemoglobin; PLT, platelet; CR, complete remission; IA, cytarabine 100 mg/m^2^ continuous infusion day1–7, idarubicin 12 mg/m^2^ day1–3; HiDAC, cytarabine 2 g/m^2^ over 3 h every 12 h on day1–3; CAG, cytarabine 10 mg/m^2^ every 12 h, day1–14; aclarubicin 5–7 mg/m^2^, daily on day1–8; and concurrent use of G-CSF 200 µg/m^2^/day; VA, venetoclax once daily (100 mg day1, 200 mg day2, 400 mg day3–28) and azacitidine 75 mg/m^2^ day1–7; allo-HSCT, allogeneic haematopoietic stem cell transplantation.
